# Non-Coding RNAs as Potential Novel Biomarkers for Early Diagnosis of Hepatic Insulin Resistance

**DOI:** 10.3390/ijms21114182

**Published:** 2020-06-11

**Authors:** Ariadna Pielok, Krzysztof Marycz

**Affiliations:** 1 Department of Experimental Biology, Wroclaw University of Environmental and Life Sciences, 50-375 Wroclaw, Poland; 2 International Institute of Translational Medicine, Jesionowa 11 St., 55-124 Malin, Poland; 3 Collegium Medicum, Cardinal Stefan Wyszyński University (UKSW), Woycickiego 1/3, 01-938 Warsaw, Poland

**Keywords:** insulin resistance, hepatic, liver, miRNA, lncRNA, ncRNA, circulating, marker

## Abstract

In the recent years, the prevalence of metabolic conditions such as type 2 Diabetes (T2D) and metabolic syndrome (MetS) raises. The impairment of liver metabolism resulting in hepatic insulin resistance is a common symptom and a critical step in the development of T2D and MetS. The liver plays a crucial role in maintaining glucose homeostasis. Hepatic insulin resistance can often be identified before other symptoms arrive; therefore, establishing methods for its early diagnosis would allow for the implementation of proper treatment in patients before the disease develops. Non-coding RNAs such as miRNAs (micro-RNA) and lncRNAs (long-non-coding RNA) are being recognized as promising novel biomarkers and therapeutic targets—especially due to their regulatory function. The dysregulation of miRNA and lncRNA activity has been reported in the livers of insulin-resistant patients. Many of those transcripts are involved in the regulation of the hepatic insulin signaling cascade. Furthermore, for several miRNAs (miR-802, miR-499-5p, and miR-122) and lncRNAs (H19 imprinted maternally expressed transcript (H19), maternally expressed gene 3 (MEG3), and metastasis associated lung adenocarcinoma transcript 1 (MALAT1)), circulating levels were altered in patients with prediabetes, T2D, and MetS. In the course of this review, the role of the aforementioned ncRNAs in hepatic insulin signaling cascade, as well as their potential application in diagnostics, is discussed. Overall, circulating ncRNAs are precise indicators of hepatic insulin resistance in the development of metabolic diseases and could be applied as early diagnostic and/or therapeutic tools in conditions associated with insulin resistance.

## 1. Introduction

In recent years, the number of people and animals suffering from conditions correlated with insulin resistance such as type 2 diabetes (T2D) and metabolic syndrome (MetS) has been rapidly rising, making them some of the most burning medical challenges of 21st century. The report of International Diabetes Federation stated that in the year 2000, the global estimate of diabetes prevalence in patients between ages 20 and 79 was 151 million, but this number tripled to a total of 463 million in 2019 [1]. By the year 2045, around 700 million people are predicted to develop diabetes. The estimated number of deaths in 2019 due to diabetes was 4.2 million, which is very alarming, especially when compared to 2016 World Health Organization (WHO) report in which diabetes was stated as a cause of death for 1.6 million patients. The existing data imply that metabolic syndrome is even more common. In most countries in the Asia-Pacific region, nearly one fifth of the adult population has been reported to be affected by MetS [2], while in the United States, the overall prevalence of MetS has fluctuated from 25.3% in 1988–1994 to 25.0% in 1999–2006, before it finally increased to 34.2% in 2007–2012 [3]. Since MetS is at least three times more common than diabetes, about 25% of world population is estimated to be affected by this disorder—which translates to over a billion people worldwide [4]. 

One of the main features of both T2D and MetS onset is insulin resistance, defined as an inability of the target tissues to orchestrate well-coordinated glucose-lowering processes such as the suppression of gluconeogenesis, lipolysis, net glycogen synthesis, and cellular glucose uptake in response to physiological insulin levels in plasma [5]. All of the above are a result of impaired insulin signaling at the cellular level. Glucose homeostasis in an organism is a state managed on multiple levels by various regulatory mechanisms. However, it is widely acknowledged that the liver, along with other tissues such as skeletal muscle and white adipose tissue, plays a crucial role in maintaining this balance [6]. The liver is recognized as the metabolic center of an organism, and, as such, it orchestrates the metabolism of carbohydrates, lipids, and proteins. In normal conditions, the liver reacts to insulin levels by facilitating the processes of glycogenesis, glycogenolysis, glycolysis, gluconeogenesis, and lipogenesis during the fasting/feeding state. However, if the hepatic insulin signaling cascade becomes impaired, as happens in the case of insulin resistance, pathologies in hepatic metabolism arise and the liver itself becomes a critical contributor to hyperglycemia, inflammation, and *de novo* lipogenesis [7,8,9]. Furthermore, mounting evidence suggests that the impairment of hepatic metabolism, resulting in hepatic insulin resistance, is a fundamental step in the development of both conditions [7,10]. The prevalence of nonalcoholic fatty liver disease (NAFLD) among T2D and MetS patients also supports this hypothesis, as 79% of MetS patients [11] and 40–60% of T2D subjects [12] are affected. Some researchers have gone even further and considered the NAFLD not only a manifestation of MetS but a crucial precursor of this condition [10]. 

Therefore, it is clear that the early diagnosis of hepatic insulin resistance could be beneficial, as it often precedes the arrival of other symptoms. However, the majority of clinically used diagnostic methods are currently based around blood glucose measurements. Metrics such as the fasting plasma glucose (FPG) value, the 2-h plasma glucose (2-h PG) value during a 75-g oral glucose tolerance test (OGTT), or HbA1C (hemoglobin A1c) criteria [13] are applied in order to determine alterations in glucose metabolism within an organism. Unfortunately, these criteria are often insufficient when performed alone, and, thus, in order to properly diagnose a patient, more than one is required. For example, according to Meijnikman et al. [14], out of a total of 581 Caucasian patients diagnosed with prediabetes, 44.2% subjects would be misdiagnosed if relying only on the HbA1c criterion without the support of the OGTT. Similar observations were made by Karnchanasorn et al. [15]. Furthermore, methods that target systemic insulin resistance have also been developed. Currently, the hyperinsulinemic-euglycemic glucose clamp technique and the frequently-sampled intravenous glucose tolerance test (FSIVGTT) [16] are recognized as the gold standards, yet their clinical application is limited [17,18]. Nevertheless, there are available methods such as homeostatic model assessment of insulin resistance (HOMA-IR), homeostatic model assessment 2 (HOMA2), the quantitative insulin sensitivity check index (QUICKI) [17], or Matsuda, which are generally accepted and considered reliable. Unfortunately, the lack of clear guidelines applicable in clinical use limits their potential for the early diagnosis of insulin resistance before system-wide alterations in carbohydrate metabolism develop. Furthermore, it has also been reported that some accompanying conditions may affect the aforementioned methods’ sensitivity and specificity. For example, in patients with polycystic ovary syndrome (PCOS), the aforementioned surrogate tests have presented a high positive predictive value (90–96%) but a low negative predictive value (36–45%); therefore, a number of subjects have remained unrecognized by any of these methods [19]. Consequently, establishing universal diagnostic methods that would target hepatic insulin resistance, which is a hallmark of prediabetes, T2D, and MetS, might be the right approach. 

Currently, a whole new group of relatively novel biomarkers is starting to resurface as early indicators of hepatic insulin resistance, namely ncRNAs (non-coding RNAs) such as lncRNA (long non-coding RNA) and miRNA (micro-RNA). Numerous lncRNAs and miRNAs have been reported to be dysregulated in the state of insulin resistance [20,21] which, when considering their crucial regulatory function, is understandable. Kornfeld et al. [22] demonstrated that in the liver of high fat diet (HFD)-mice, the expression of 66 miRNAs was significantly altered; from which the expression of 90.1% of genes was increased and only 9.1% was decreased. Additionally, in the livers derived from Lepr db/db (homozygous for the diabetes db mutation of the leptin receptor) mice, the expression of 156 miRNAs was altered when compared to healthy controls. 

The bioinformatics analysis performed by Yuan et al. [21] presented even more impressive data, as 4614 (2719 up-regulated and 1893 down-regulated) hepatic miRNAs and 2813 (818 up-regulated and 1995 down-regulated) lncRNAs were dysregulated in the livers of HFD male Sprague Dawley rats. Therefore, it has become clear that those non-coding RNAs possess an important diagnostic potential for the detection of hepatic insulin resistance. Especially since published evidence has suggested that both miRNAs and lncRNAs serve as very inclusive and sensitive markers among patients with varying ethnicities or medical conditions. For example, the circulating levels of the lncRNA metastasis associated lung adenocarcinoma transcript 1 (MALAT1) were found to be increased in women with GDM (gestational diabetes mellitus) [23]. However, the value of a marker partly lies in the possibility of its easy acquisition for the purpose of further testing. Therefore, among the plethora of hepatic dysregulated miRNAs and lncRNAs, those in which circulating levels correlate with the hepatic state are of special interest because collecting a single blood sample from a patient is a clinically achievable practice performed on a daily basis.

In this review, the chosen miRNAs, including *miR-802, miR-499-5p, and miR-122-5p*, and lncRNA, including maternally expressed gene 3 (*MEG3*), *MALAT1*, and H19 imprinted maternally expressed transcript (*H19*) are discussed. Additionally, the prognostic potential of the described ncRNAs in circulation is addressed. The aforementioned ncRNAs were chosen because they play a crucial role in the regulation of the hepatic insulin signaling cascade, and, therefore, their hepatic expression patterns are significantly altered in subjects affected by hepatic insulin resistance. Furthermore, the levels of described ncRNAs in circulation vary in accordance with similar fluctuations in the liver, which makes them promising biomarkers in terms of future use in early hepatic insulin resistance diagnostics (Table 1). 

## 2. Hepatic Insulin Signaling Cascade

**Figure 1:**
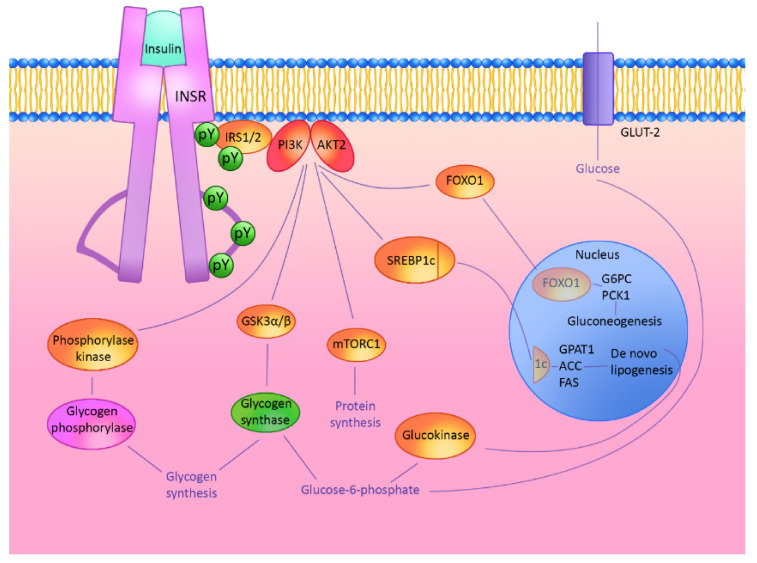
Hepatic insulin signaling cascade.

The liver is referred to as the metabolic center of the organism, and, as such, it plays a pivotal role in glucose homeostasis, along with white adipose tissue and skeletal muscles [5]. Within the hepatic tissue, insulin facilitates a number of different reactions, such as the reduction of hepatic glucose production and the promotion of glycogen synthesis. Additionally, it also affects the synthesis of numerous lipids and proteins including albumin and fibrinogen [45,46]. The insulin signaling cascade in hepatocytes begins in a manner universal to all cells—by binding to an INSR (insulin receptor). As a result of this binding, the INSR is activated. Next, the recruitment of various scaffold proteins—of which the insulin receptor substrate (IRS) family remains as the best-studied—occurs (Figure 1). IRS1 and IRS2 (insulin receptor substrate 1 and 2) are the two main isoforms expressed in hepatocytes. Pi3K (phosphatidylinositol 3-kinase) is recruited by the IRS and catalyzes the production of PIP3 (prolactin induced protein 3) from PIP2 (prolactin induced protein 2), which then binds to Akt (protein kinase B). Simultaneously, insulin inhibits PTEN (phosphatase and tensin homolog) because it catalyzes a reverse reaction that enables the accumulation of PIP3. Subsequently to the PIP3 binding, Akt is activated by the phosphorylation of motifs in its activation loop—Thr308 and Ser473, in Akt1. The diversity of Akt substrates indicates that the ramifications of the insulin signaling pathway appear subsequently to Akt. FOXO (forkhead box), GSK3 (glycogen synthase kinase 3), or various regulators of mTOR (mechanistic target of rapamycin kinase) activity are among Akt substrates. FOXO1 (forkhead box O1) regulates gluconeogenic gene transcription, while GSK3 regulates glycogen synthesis. Upon activation, Akt phosphorylates FOXO1, which results in FOXO1’s exclusion from nucleus, where it regulates the activity of G6PC (glucose-6-phosphatase catalytic subunit) and PCK1 (phosphoenolpyruvate carboxykinase 1)—two genes involved in hepatic gluconeogenesis. Upon entering the cytosol, FOXO1 undergoes deactivation via ubiquitination. In addition to the glucose metabolism, insulin also regulates lipids anabolism and catabolism within the liver, particularly by the SREBP-1c (sterol regulatory element-binding proteins) signaling pathway. Since insulin promotes net hepatic lipogenesis in standard conditions, insulin-resistant patients should be characterized by decreased lipogenesis, and such a phenotype was described in mice with total genetic insulin resistance (ablation of the hepatic insulin receptor) [47]. However, in normal humans and rodents, an opposite effect has been observed because insulin resistance is usually accompanied by hepatic steatosis and increased net hepatic lipogenesis, often resulting in NAFLD development. This anomaly is known as “pathway-selective insulin resistance and responsiveness” [5,48,49,50]. Conclusively, insulin resistance imposes a vast and diverse effect on hepatic metabolism and the liver’s ability to maintain glucose and lipid homeostasis.

## 3. microRNAs and lncRNAs—Crucial Regulators of Cellular Pathways

miRNAs are a large group of small (15–22 nts), non-coding sequences with hairpin conformation, highly conserved among the species. Since their discovery 20 years ago, their biogenesis, activity, and function have been intensively studied and paved the path for much revolutionary research. However, their importance has been long underestimated due to technological limitations. It was not until the discovery that two miRNAs—*lin-4* and *let-7*—control the timing in nematode (*Caenorbabditis elegans*) development that the significance of miRNA was fully recognized [51]. At this point, miRBase, which is an online miRNA database, lists 2654 mature miRNAs and 1917 precursor miRNAs (pre-miRNAs) in *Homo sapiens*. Simultaneously, around 60% of protein-coding human genes possess predicted miRNA target sites [52]. 

The dysregulation of miRNAs has been described in numerous diseases [53], including T2D, MetS, and NAFLD. miRNA expression tends to be largely tissue-specific and transcriptionally regulated. Usually, they are located within introns or lncRNAs, and their transcription is facilitated by RNA-polymerase. Importantly, miRNAs are organized into families based on the similarity of their seed sequences, which are two-to-eight nucleotides long (starting from the 5′ end) and determine the targeted mRNAs. Consequently, the main role of miRNA concerns the posttranscriptional regulation of mRNA expression via the destabilization or repression of the transcript [52]. This function makes miRNA a part of virtually any cellular process—including development determination, metabolism, cell activity, and apoptosis. One miRNA may target multiple genes, while one gene can be regulated by a number of miRNAs, which clearly underlines miRNAs’ critical roles as regulators—especially since entire signaling pathways might be controlled by either clusters of miRNAs or a single miRNA [52]. In turn, the role and biological function of recently discovered lncRNAs (over 200 nts) is still not fully elucidated. However, the cross-talk between these two types of non-coding RNA clearly indicates that lncRNA has the potential to affect miRNA activity. 

lncRNAs are loosely categorized together in a very diverse and large group, and, similarly to miRNA, their input in the regulation of numerous biological processes is crucial. They have been reported to partake in genomic loci imprinting, allosterically regulating enzymatic action or even chromosome conformation [54,55]. Intricate regulatory lncRNA patterns have been described in a variety of cellular mechanisms, and their dysregulation has also been demonstrated in different disorders [56]. In general, lncRNAs can be categorized into three broad subgroups based on their function. First, the non-functional lncRNAs, which are probably a product of transcriptional noise. The second comprises lncRNAs, the transcripts of which are of lesser importance and the act of transcription itself is sufficient for their action. Finally, the third group consists of the functional lncRNAs with the ability to act in *cis* and/or *trans* orientations [57]. In some aspects, lncRNAs are similar to mRNAs, as they are transcribed by polymerase II from the genomic loci, often 5′-capped, spliced, and polyadenylated. Some lncRNAs can even produce small peptides [58]. Among the wide repertoire of lncRNAs functions, they also pose the ability to interact with miRNAs in various ways. It has been described that miRNAs can target lncRNAs in order to reduce their stability. Furthermore, lncRNAs can act as molecular sponges or decoys of miRNAs and, consequently, regulate the miRNA level in the cytoplasm by binding specific miRNAs, and actively sequestrate them from their target mRNAs; such lncRNAs are defined as competing endogenous (ce) RNAs [59,60]. Moreover, lncRNAs can also compete with miRNAs for binding sites in shared target mRNAs [61]. Therefore, next to miRNAs, lncRNAs form another viable group of highly-specialized regulators of gene expression, and both of these ncRNAs have been reported to act as regulators of the hepatic insulin signaling cascade [62,63]. 

Consequently, *miR-499-5p* has been described to regulate *PTEN* expression, while *miR-802* and *MEG3* are involved in gluconeogenesis. Some ncRNAs can even affect more than one ramification of the insulin signaling pathway like *miR-122, MALAT1*, and *H19*, which are also correlated with hepatic *de novo* lipogenesis. 

### 3.1. miR-802

microRNA-802, located on the 21st chromosome, has been intensively studied in different types of cancer, and it has been shown that it plays a crucial role during metastasis, progression, and invasion. Huang et al. [64] observed that *miR-802* not only inhibits the proliferation and invasion but also the epithelial–mesenchymal transition of glioblastoma multiforme cells. *miR-802’s* role has also been described in tongue squamous cell carcinoma [65], pancreatic cancer progression [66], and human cervical cancer [67]. Recently, Zhang et al. [68] demonstrated *miR-802’s* ability to inhibit the aggressive behaviors of non-small cell lung cancer cells. However, it has also been described that *miR-802* can accelerate the growth of hepatocellular carcinoma (HCC) [69] and that its high concentration in blood is associated with a poor prognosis in HCC patients [70]. Furthermore, *miR-802* has been shown to partake in obesity-induced nephropathy in obese human and mice [71]. In fact, *miR-802* upregulation associated with obesity and insulin resistance has been observed in different tissues such as the kidney, white adipose tissue, skeletal muscle, and the liver [24]. As is widely known, the damage occurring in target organs during diabetes is multicausal, so next to high glucose and fatty acids levels, the increased production of reactive oxygen species (ROS) also accounts for the damage. Yang et al. [25] demonstrated a connection between *miR-802*, oxidative stress, and hepatic insulin resistance. In their study, they showed that in HFD mice, *miR-802* upregulation was associated not only with higher blood glucose and serum insulin levels but also with a lowered activity of oxidative-stress related enzymes such as SOD (superoxide dismutase), CAT (catalase), and GSH-Px (glutathione peroxidase). Following this thread, ROS generation was significantly greater upon *miR-802* upregulation and could be improved by *miR-802* inhibition. Subsequently, the expression levels of key proteins in the insulin signaling cascade were investigated in order to assess the influence of *miR-802’s* up-regulation of hepatic insulin resistance. Interestingly, the expression of phosphorylated Akt1 was decreased, while the phosphorylated IRS1 (Ser307) level was increased. Conspicuously, IRS1 phosphorylation in the Ser307 site was found to result in the decreased phosphorylation of IRS-1 tyrosine residues, which, in turn, leads to the accelerated degradation of this protein and (as a consequence) the reduced phosphorylation of its target—Akt [72]. The vital role of *miR-802* upregulation in hepatic insulin resistance was also supported by Kornfeld et al. [22]. In their study, the up-regulation of *miR-802* in the liver was once again confirmed in both HFD mice and Lepr db/db mice. Furthermore, this up-regulation was shown to negatively affect *Hnf1b* (hepatocyte nuclear factor 1-beta) expression, which consequently resulted in the induction of *PPARγ* (peroxisome proliferator-activated receptor gamma coactivator) and its target genes: *G6pc* (glucose-6-phosphatase catalytic subunit) and *Pck1* (phosphoenolpyruvate carboxykinase 1), both involved in gluconeogenesis. Similar findings were described by Higuchi et al. [24], who also demonstrated that *miR-802* circulating levels were significantly increased in patients with T2D; therefore, *miR-802* could be used as a marker for T2D.

### 3.2. miR 499-5p

The microRNA-499 gene is located in the 20th intron of the *Myh7b* (beta-myosin heavy chain 7B) [73], and, therefore, it is often associated with cancer risk because it might affect *Myh7b* gene function. It has also been reported to downregulate a proto-oncogene *ETS1* in HepG2 cells (human liver hepatocellular carcinoma cells) by increasing *MMP-7* (matrix metallopeptidase 7) expression [74]. As was observed in a study by Ma et al. [75], *miR-499* polymorphism is also connected with a higher susceptibility to hepatocellular carcinoma in a large-scale population. Furthermore, *miR-499* genetic variation has also been reported as a crucial indicator of diabetic neuropathy susceptibility and could indicate patients with a higher risk of developing cardiovascular autonomic neuropathy [76]. Recently, a link between *miR-499* and NAFLD was also discovered, as it was demonstrated by Hanyun et al. [77] that *miR-499* inhibition improves NAFLD which, as was previously mentioned, is often associated with insulin resistance. The experimental model involved specific pathogen-free (SPF) male C57BL/6 mice fed with a high-fat diet and injected with an *miR-499* inhibitor. Subsequently, it was observed that the degree of steatosis in the liver tissue of the mice treated with the *miR-499* inhibitor was significantly lower that of the untreated control group. Furthermore, compelling evidence that *miR-499* is in fact involved in hepatic glucose metabolism was presented by Wang et al. [26], who observed that *miR-499* levels were significantly lower in the livers of db/db and HFD-fed mice. Interestingly, Akt/GSK activation impairment was observed alongside *miR-499-5p* downregulation. By applying bioinformatics tools followed by in vitro testing, it was revealed that *miR-499* affects the insulin signaling cascade and glycogen synthesis by suppressing *PTEN*. This observation was further confirmed, because introducing *miR-499* into HFD mice resulted in *PTEN* suppression and the improvement of Akt/GSK activation. The association between *miR-499* levels and T2D was also described in the erythrocytes of African-American adults [27], as it was observed that *miR-499* levels were reduced in pre-diabetic patients. Furthermore, a strong correlation between *miR-499* and HbA1c criterion was visible, which is especially promising in terms of its possible use in diagnostics.

### 3.3. miR 122-5p

*miR-122* is located on human chromosome 18 and was one of the first identified factors of so-called tissue-specific miRNA [78], as it is highly abundant in the liver, in which it is recognized as the dominant miRNA that accounts for 70% of hepatic miRNA content in mice and about 52% of human hepatic miRNome [79]. Moreover, *miR-122* is strongly conserved among vertebrate species, which indicates its crucial function. *miR-122* is highly expressed, particularly in adult livers, as it expression increases during embryonic development. Consequently, *miR-122*, under the regulation of *HNF6* (hepatocyte nuclear factor 6) and *OC2* (one cut homeobox 2), partakes in the terminal differentiation of hepatocytes, as demonstrated by Laudadio et al. [80]. Additionally, the inhibition of *miR-122* activity in BMEL (bipotent murine embryonic liver) cells cultured as floating aggregates led to a repressed expression of 26 genes, 24 of which were coding hepatocyte-specific proteins, while the expression of 22 hepatic non-specific genes was increased. Therefore, it is clear that *miR-122* participates in the differentiation of hepatoblasts towards hepatocytes; however, its activity also extends towards other processes. As was previously described, *miR-122* plays a pivotal role in regulating hepatic gene expression, affecting various aspects of cellular activity such as response to oxidative stress [81], viral infection [82], inflammation [83], and even tumorigenesis, as its dysregulation has notoriously been reported as an viable marker of HCC onset and development [84,85]. *miR-122* has also been described to orchestrate lipid metabolism; as Cheung et al. [28] demonstrated in their study, *miR-122* inhibition in healthy mice resulted in a decreased expression of hepatic *de novo* lipogenesis genes such as *FASN* (fatty acid synthase) and *ACC1* (acetyl-CoA carboxylase), which encode two rate-limiting enzymes. Furthermore, the inhibition of *miR-122* diminished hepatic lipogenesis and served as a protection against liver steatosis in HFD obese mice. Additionally, it was described by Iliopoulos et al. [29] that *miR-122* overexpression in HepG2 cells also resulted in increased *SREB-1c* expression along with the aforementioned *ACC-1* and *FASN*. The culminating evidence indicated a critical role of *miR-122* in hepatic *de novo* lipogenesis regulation and, in consequence, NAFLD development, a common condition among patients affected by either MetS or T2D. Additionally, *miR-122* dysregulation has been shown to accompany hepatic insulin resistance. Dong et al. [30] described how—in both a T2D rat model and an IR HepG2 cell culture—*miR-122* expression was significantly increased. Furthermore, *miR-122* overexpression resulted in a decreased expression of its target: insulin-like growth factor (*IGF-1R*), which, in turn is a part of the IGF-1R/PI3K/Akt signaling pathway. This negative effect could be reversed by *miR-122* inhibition. miR-122 has also been shown to affect the hepatic gluconeogenesis process. In a study published by Wei et al. [31], it was demonstrated that *HNF-4α* (hepatocyte nuclear factor 4 alpha) acts as a regulator of *miR-122* activity, which, in turn, regulates a plethora of targets associated with gluconeogenesis (*G6PC* and *PCK1*) and *de novo* lipogenesis (*SREBP-1c* and *FAS*). Furthermore, compelling evidence regarding the *miR-122* level’s prognostic value was demonstrated by Willeit et al. [32]. Specifically, it was showcased that *miR-122’s* circulating levels were significantly higher among subjects with insulin resistance. Patients suffering from MetS were characterized by 160% higher circulating *miR-122* levels, while T2D subjects displayed 240% higher levels when compared to a control group; *miR-122* was also recognized as an indicator of future MetS and T2D onset. Taking all supporting evidence into account, *miR-122* seems as a very promising and well established marker of hepatic insulin resistance with a great prognostic value.

### 3.4. Long-Non-Coding RNA MALAT1(Metastasis Associated in Lung Adenocarcinoma Transcript 1)

As one of the first long non-coding RNAs to be discovered, *MALAT1* is very well-conserved among different mammal species. Additionally known as noncoding nuclear-enriched abundant transcript 2 (*NEAT2*), it is located on the short arm of human chromosome 11q13.1 and was has been shown to take a part in regulation of cells proliferation and motility. *MALAT1* is also highly abundant in various organs. Furthermore, the bioinformatics analyses performed by Chen *et al* [86] indicated that *MALAT1* regulates the expression of many distinct genes by affecting various stages of their transcription and elongation. Repeatedly, its dysregulation has been observed in a wide range of human cancers such as osteosarcoma, breast cancer, uterine endometrial stromal sarcoma, cervical cancer, hepatocellular carcinoma, and colorectal cancer [87]. However, bearing in mind the diverse ramifications of *MALAT1* action, it has also been linked with viral infections, stimulating cytokine production and even alcohol abuse. Recent evidence has indicated *MALAT1’s* effect on glucose and lipid metabolism [88]. Several studies have discussed its involvement in diabetes and diabetic-complications. In 2019, Puthanveetil et al. [88] observed that maintaining human umbilical vein endothelial cells (HUVECs) in high-glucose conditions led to the overexpression of *MALAT1*, which, in turn, induced *IL-6* (interleukin-6) and *TNF-α* (tumor necrosis factor α) up-regulation via *SAA3* (serum amyloid A3), one of *MALAT1*’s targets. Subsequently, this *MALAT1* activity was confirmed in renal tissue obtained from diabetic mice. Yan et al. [89] provided evidence that *MALAT1* overexpression in the endothelial cells of mice was associated with diabetes. Following this thread, Yan et al. investigated the expression of *MALAT1* in two models of diabetes: the livers of ob/ob mice and hepatocytes exposed to palmitate, and they were able to prove that *MALAT1* expression was significantly up-regulated in both models. Furthermore, it was confirmed that *MALAT1* overexpression led to the up-regulation of mRNA and nuclear *SREBP-1c. SREBP-1* is a major regulator of *de novo* lipogenesis, so its up-regulation is strongly correlated with liver steatosis—a main symptom of NAFLD. Non-alcoholic fatty liver disease is highly prevalent among patients affected by metabolic syndrome—it is often referred to as the manifestation of metabolic syndrome with a prevalence reaching up to 79% [11]. NAFLD is also highly abundant in type 2 diabetes patients (40–60%) [12], as clearly visible insulin resistance affects not only the glucose metabolism but also lipids. Furthermore, it has been repeatedly reported that oxidative stress is highly correlated with insulin resistance and can also be the cause of poor insulin sensitivity. Chen et al. [86] provided evidence of how *MALAT1* is involved in oxidative stress-mediated insulin resistance via the up-regulation of the *Jnk* (c-Jun N-terminal kinase)—a stress-sensitive kinase that, upon activation, can suppress insulin signaling by inhibiting the phosphorylation of IRS and Akt—two major regulators in the insulin signaling cascade. The difference in the expression of *MALAT1* was also observed in GDM patients [23]. By measuring the plasma levels of three long non-coding RNAs, Zhang et al. were able to prove that *MALAT1* levels were higher in patients with GDM when compared to healthy controls. The mentioned study is especially promising in terms of the future utilization of lncRNAs as easily-acquired and sensitive markers for insulin resistance detection.

### 3.5. Long-Non-Coding RNA MEG3 

The lncRNA *MEG3* is characterized as an imprinted gene that belongs to the imprinted delta like non-canonical notch ligand (*DLK1-MEG3*). In humans, it is located on the 14q32.3 chromosome, while in mice, it is known as gene trap locus 2 (*Gtl2*) and is located at distal chromosome 12. *MEG3* expression if often affected in various types of cancer. Up to this point, the loss of its expression has been described in gastric cancer, gallbladder cancer, non-small cell lung cancer (NSCLC), and cervical cancer [33]. *MEG3* is sometimes referred to as tumor suppressor because its overexpression promotes apoptosis and inhibits the proliferation of tumor cells. In the recent years, mounting evidence has also revealed *MEG3’s* connection to type 2 diabetes and insulin resistance in the pancreas [34], diabetic microvascular dysfunction [35], and nephropathy [36]. Additionally, the overexpression of *MEG3* has been observed in the livers of ob/ob mice and HFD-induced insulin-resistant mice, ultimately resulting in the disruption of the insulin signaling cascade through the upregulation of *Foxo1* [37]. As *Foxo1* regulates the activity of *G6pc* and *Pck1*, this action of *MEG3* promotes hepatic gluconeogenesis. After further exploring the subject, Zhu et al. [90] proposed a possible connection between *FOXO1* and *MEG3* because the later participates in regulating *ATF4* (activating transcription factor 4) expression—one of *FOXO1’*s co-regulators—by acting as a sponge for *miR-214*, which is known as an *ATF4* suppressor. As was shown in recent years, lncRNA may competitively bind miRNA and, by doing so, act as a ceRNA (competing endogenous RNA). A similar mechanism was described by Chen et al. [38], who observed the upregulation of *MEG3* in two experimental models: HFD mice and palmitate-treated hepatocytes serving as models of insulin resistance. In this instance, the expression of *miR-185-5p* and its target mRNA-*EGR2* (early growth response 2) was evaluated. Again, *MEG3* acted as a ceRNA for *miR-185-5p* and, as a consequence, promoted the expression of *EGR2*—which was reported to inhibit IRS and partake in fat cell differentiation. The expression of MEG3 was also studied in PBMCs (peripheral blood mononuclear cells), and, again, its overexpression was showcased in patients affected by type 2 diabetes [39].

### 3.6. Long-Non-Coding RNA H19

Similarly to *MALAT1*, *H19* was one of the first long-non-coding RNAs to be discovered, and it has consequently been thoroughly studied. It is located on human chromosome 11 or on chromosome 7 in mice, and it is comprised of five exons and four introns. The biological functions of *H19* are quite versatile, as it partakes in the regulation of cells proliferation and differentiation processes [91]. It has been reported to act as a tumor suppressor but also as an oncogene. Furthermore, it plays a pivotal role in proper embryonic development because it regulates various important genes such as *IGF2* that belong to the imprinted gene network (*IGN*). However, after embryonic development occurs, its action in limited to only few tissues such as heart and skeletal muscles. In a healthy adult liver, *H19* has been detected on very low yet appreciable levels; however, Nilsson et al. observed that in adults with type 2 diabetes, *H19* hepatic levels were elevated [40]. Zhang et al. [41] provided evidence of a similar *H19* expression pattern in HFD mice. Furthermore, by performing *H19* knock-down in HepG2 cells followed by an RNA-seq analysis and qPCR, Zhang observed a decreased expression of *HNF4A*, as well as *PCK1* and *G6PC*—two major gluconeogenic genes. In turn, the overexpression of *H19* was accompanied by an increased expression of those genes. Therefore, the mechanism by which *H19* is involved in hepatic glucose homeostasis seems to be strongly associated with excessive hepatic glucose production mediated by *H19’s* ability to regulate *HNF4A* methylation. Interestingly, by using a fasting mouse model, Zhang et al. were able to demonstrate that the *H19* upregulation might be a physiological response to fasting aimed at restoring glucose homeostasis by elevating hepatic glucose production. However, contradictory evidence has also been published. Goyal et al. [42] demonstrated that in the livers of db/db mice, *H19* was downregulated and that this alteration was correlated with *Foxo1* upregulation and its nuclear retention, which consequently led to the increased expression of *G6pc* and *Pck1*. Furthermore, Goyal et al. confirmed this mechanism of *H19*’s regulatory activity by treating C57BL/6J mice with an H19 inhibitor [42]. These conflicting findings may possibly be explained by the fact that each study was based on a different in vivo model. Liu et al. [43] observed *H19* up-regulation, *Srebp-1c, Acc1, Scd1, Fasn and Pparγ*, increased expression in a model of NAFLD. This study was also conducted based on HFD mice model, so it is possible that *H19* expression varies between different models, especially since lncRNA expression patterns are tissue- and state-specific. Nevertheless, further research might be needed to provide the answer. Conclusively, evidence presented by Liu et al. further underlined the magnitude of *H19*’s input in hepatic insulin resistance development because it affects not only gluconeogenesis but also hepatic lipogenesis. Recently, Fawzy et al. [44] found that *H19* circulating levels were significantly increased among patients with T2D, which indicates that it may serve as a blood-based marker of hepatic insulin resistance, especially since its dysregulation has been described in a variety of metabolic disorders.

## 4. Prognostic Potential

Mounting evidence published in recent years has indicated that ncRNAs could be applied to developing specific and sensitive diagnostic methods. Numerous publications have described both miRNAs and lncRNAs as precise markers of various diseases. Nevertheless, there are some practical issues that need to be addressed in order to maximize ncRNAs’ utility as markers applicable for every day clinical use. As pointed out in various studies, monitoring a single ncRNA’s expression is often insufficient to grasp a full and precise diagnosis. The majority of literature published in the field has clearly suggested that establishing a panel of ncRNAs is the most efficient approach. The discussed ncRNAs could be potential candidates for such a panel. As Higuchi et al. [24] demonstrated in their study, circulating levels of *miR-802* are visibly correlated with HbA1c, HDL-C (high density lipoprotein cholesterol), and estimated glomerular filtration rate test eGRF values and, as such, could be used as a biomarker of T2D with MetS. Additionally, Church et al. [92] conducted a study in which hepatobiliary injury and biliary hyperplasia were inflicted in rats with alpha-naphthylisothiocyanate (ANIT) and a proprietary compound, FP004BA. Such a treatment resulted in hepatocellular necrosis and the enrichment of various ingenuity pathways, some of which were also altered during hepatic insulin resistance. At 24 h after the oral administration of ANIT, pathways related to cholesterol biosynthesis and oxidative stress response were among the most affected, while 120 h after administration, glycogen and oleate biosynthesis canonical pathways were enriched. Subsequently, Church et al. demonstrated that among 60 assessed miRNAs, *miR-802* was the most elevated during hepatobiliary injury and, as such, was a precise marker of liver injury. Additionally, even in case of slight hepatocellular injury, a trend towards an *miR-802* level increase was observed. Bearing in mind the aforementioned findings, *mir-802* should be considered as a valuable marker of hepatic distress and insulin resistance. Up to this point, *miR-499-5p* has been strongly associated with cardiovascular pathologies [93,94,95,96]; however, evidence regarding its diagnostic potential in diabetic and prediabetic patients has also been published. As Fluitt et al. [27] demonstrated, *miR-499-5p* was significantly reduced in erythrocytes of African American pre-diabetic patients. Additionally, it could be applied to effectively distinguish prediabetic patients from those with T2D (area under the curve (AUC) = 0.7866; *p* = 0.02). A similar expression pattern of *miR-499-5p* was also observed among diabetic ESRD (end-stage renal disease patients) undergoing dialysis [97] and in patients affected by diabetic neuropathy or diabetic polyneuropathy [76]. These findings clearly indicate that *miR-499-5p* is a precise marker in the course of T2D and its complications. Collectively, with its role in hepatic insulin resistance development, *miR-499-5p* could be a potential candidate for developing a diagnostic panel of ncRNAs that are applicable in clinical use. As the most abundant hepatic miRNA, *miR-122* is a well-established marker of liver distress. As Jampoka et al. [98] demonstrated in their study, *mir-122* was an efficient, serum-derived marker of NAFLD, with 75% sensitivity and 82.35% specificity(the AUC was 0.831; *p* < 0.0001). What is more, there was a significant difference in *miR-122* serum levels between patients without steatohepatitis and those affected by it. Therefore, *miR-122* was not only a precise marker of NAFLD but could also be applied to assess the severity of hepatic damage. Furthermore, *miR-122* plasma levels could precisely indicate T2D patients who develop NAFLD [99]. As similar observations have been published by different authors [79,100], the collective body of evidence clearly highlights *miR-122’s* hepatic specificity and diagnostic potential. Since lncRNAs are not as well studied as miRNAs, the number of potential lncRNA markers is still growing. However, some lncRNAs have already been established as good indicators of insulin resistance and/or hepatic distress. *MALAT1* has been reported as an important agent in hepatic insulin resistance development. As Konishi et al. [101] described in their study, *MALAT1* plasma levels were associated with liver damage and HCC development. Similar evidence has been published by other authors [102]. Additionally, *MALAT1* has been proven as an acceptable marker of GDM, as Zhang et al. demonstrated in their study that the AUC was 0.654 (95% confidence interval 0.543–0.768), with a 50% sensitivity and an 83% specificity [23]. Unfortunately, up to this point, no data concerning the specificity and sensitivity of circulating *MEG3* in cases of hepatic insulin resistance have been published. However, such analyses have been performed for other hepatic disorders such as fibrosis, hepatitis B virus (HBV) infection [103], and HCC [104]. It is important to point out that in all the aforementioned disorders, the expression pattern was different from the one observed in T2D patients, i.e., the hepatic expression and/or circulating levels were decreased. Bearing in mind that *MEG3* was overexpressed in the PBMC of T2D patients [39] and was proven as an valuable marker in other hepatic disorders, it is clear that is should be considered a candidate for a diagnostic panel. Similarly, *H19* was described as a significant prognostic marker in chronic liver disease patients with or without cirrhosis and HCC [105]. Consequently, as Fawzy et al. [44] demonstrated in their study, *H19* levels are significantly higher in plasma of T2D patients, though a receiver operating characteristics (ROC) analysis was not performed. Additionally, Tello-Flores et al. [106] demonstrated in their study that the relative expression of serum *H19* was two-fold higher among patients with impaired glycemic control when compared to diabetics with proper glucose control. Furthermore, an association between T2D susceptibility and genetic variants of both *H19* and *MEG3* was demonstrated [107]. Nevertheless, further investigation is needed to fully elucidate the prognostic potential of *H19*. In summary, even though in some cases there have been no data describing the prognostic value of the described ncRNAs in the course of hepatic insulin resistance, due to the existing body of evidence indicating their strong affiliation with hepatic disorders and T2D, they are worth considering for diagnostic purposes.

## 5. Summary and Future Perspectives

According to the International Diabetes Federation and WHO reports, diseases associated with insulin resistance such as T2D, MetS, and NAFLD are become progressively more prevalent among the global population and will become a significant challenge in the coming years. Such a situation calls for immediate, reliable solutions and early, precise diagnosis is undoubtedly one of them. Endocrinology, as a constantly evolving field of medicine, has abandoned the idea of single-causal disease mechanisms, and this perspective has since been replaced by a more comprehensive, broader approach. Therefore, the importance of cellular metabolism alterations in the development of diseases is recognized as equal to other components such as diet or environment. The extensive research of insulin signaling pathways and the occurring pathologies accompanying metabolic diseases has indicated the significance of hepatic insulin resistance. It is recognized as one of the major agents in the development of hyperglycemia and dyslipidemia in the course of T2D and MetS. This statement is supported by evidence showcasing how targeting hepatic metabolism leads to overall clinical improvement in insulin-resistant subjects [108,109,110]. Along with the discovery of non-coding RNAs, a whole new layer of molecular mechanisms and gene expression regulation has unraveled. RNAs’ unique regulatory function links them with virtually all cellular processes, and the dysregulation of miRNAs and lncRNAs has also been described alongside various liver metabolism alterations, insulin resistance among them. Their tissue-specific expression patterns give a valuable insight into particular tissue metabolisms, which further adds to their diagnostic potential. Consequently, both lncRNAs and miRNAs have emerged as precise and versatile potential biomarkers of numerous disorders such as Alzheimer’s disease [111], breast cancer [112,113], gastric cancer [114], cardiovascular disease [95], and postmenopausal osteoporosis [115]. Additionally, due to their unique regulatory functions, the analysis of lncRNAs’ and miRNAs’ dysregulation has expanded our understanding of various conditions and their pathomechanisms. Therefore, miRNAs and lncRNAs are now being considered as potential therapeutic targets [116], not only due to the aforementioned regulatory function but also because of their mutual interactions that allow for precise intervention in cellular processes. Furthermore, the abundant presence of ncRNAs in body fluids allows for their use as precise blood based biomarkers, which indicates their potential in everyday clinical practice. In summary, both miRNAs and lncRNAs exhibit features that make them promising tools in early hepatic insulin resistance diagnosis, so their incorporation into clinical practice would be step forward towards holistic and personalized medicine.

## Figures and Tables

**Table 1:** Circulating non-coding RNA (ncRNAs) altered in hepatic insulin resistance.

ncRNA	Status in Liver	Status in Circulation	Reference
*miR-802*	↑	↑	[22,24,25]
*miR-499-5p*	↓	↓	[26,27]
*miR-122-5p*	↑	↑	[28,29,30,31,32]
*lnc MEG3*	↑	↑	[33,34,35,36]
*lnc MALAT1*	↑	↑	[23,37,38,39]
*lnc H19*	↑/↓ *	↑	[40,41,42,43,44]

* Findings differ between various research.

## Abbreviations

MetS	Metabolic Syndrome
T2D	Type 2 Diabetes
NAFLD	Nonalcoholic Fatty Liver Disease
HCC	Hepatocellular Carcinoma
WHO	World Health Organization
FPG	Fasting Plasma Glucose
2-h PH	2-h Plasma Glucose
OGTT	Oral Glucose Tolerance Test
HbA1C	Hemoglobin A1c
FSIVGTT	Frequently-Sampled Intravenous Glucose Tolerance Test
HOMA-IR	Homeostatic Model Assessment of Insulin Resistance
HOMA2	Homeostatic Model Assessment 2
QUICKI	Quantitative Insulin Sensitivity Check Index
PCOS	Polycystic Ovary Syndrome
ncRNA	Non-Coding RNA
lncRNA	Long Non-Coding RNA
pre-miRNA	Precursor miRNA
miRNA	microRNA
(ce)RNA	Competing Endogenous RNA
Nts	Nucleotides
mRNA	Messenger RNA
MALAT1	Metastasis Associated Lung Adenocarcinoma Transcript 1
MEG3	Maternally Expressed Gene 3
H19	H19 Imprinted Maternally Expressed Transcript
HFD-mice	High Fat Diet Mice
Lepr db/db	Homozygous for the Diabetes db Mutation of the Leptin Receptor
GDM	Gestational Diabetes Mellitus
INSR	Insulin Receptor
IRS	Insulin Receptor Substrate
IRS1	Insulin Receptor Substrate 1
IRS2	Insulin Receptor Substrate 2
Pi3K	Phosphatidylinositol 3-kinase
PIP3	Prolactin Induced Protein 3
PIP2	Prolactin Induced Protein 2
Akt	Protein Kinase B
PTEN	Phosphatase and Tensin Homolog
FOXO	Forkhead Box
FOXO1	Forkhead Box O1
GSK3	Glycogen Synthase Kinase 3
mTOR	Mechanistic Target of Rapamycin Kinase
G6PC	Glucose-6-Phosphatase Catalytic Subunit
PCK1	Phosphoenolpyruvate Carboxykinase 1
SREBP-1c	Sterol Regulatory Element-Binding Proteins
ROS	Reactive Oxygen Species
SOD	Superoxide Dismutase
CAT	Catalase
GSH-Px	Glutathione Peroxidase
Hnf1b	Hepatocyte Nuclear Factor 1-beta
PPARγ	Peroxisome Proliferator-Activated Receptor Gamma Coactivator
Myh7b	Beta-Myosin Heavy Chain
MMP-7	Matrix Metallopeptidase 7
SPF	Specific Pathogen-Free
HNF6	Hepatocyte Nuclear Factor 6
OC2	One Cut Homeobox 2
BMEL	Bipotent Murine Embryonic Liver Cells
FASN	Fatty Acid Synthase
IR	Insulin-Resistant
Acc1	Acetyl-CoA Carboxylase
HNF-4α	Hepatocyte Nuclear Factor 4 α
NEAT2	Noncoding Nuclear-Enriched Abundant Transcript 2
HUVECs	Human Umbilical Vein Endothelial Cells
IL-6	Interleukin-6
TNF-α	Tumor Necrosis Factor α
SAA3	Serum Amyloid A3
JNK	c-Jun N-Terminal Kinase
DLK1-MEG3	Imprinted Delta Like Non-Canonical Notch Ligand
GTL2	Gene Trap Locus 2
NSCLC	Non-Small Cell Lung Cancer
ATF4	Activating Transcription Factor 4
EGR2	Early Growth Response 2
PBMC	Peripheral Blood Mononuclear Cells
IGF2	Insulin-Like Growth Factor 2
IGN	Imprinted Gene Network
RNA-seq	RNA Sequencing
SCD1	Stearoyl-CoA Desaturase
HDL-C	High-density Lipoprotein Cholesterol
eGRF	Estimated Glomerular Filtration Rate Test
ANIT	Alpha-Naphthylisothiocyanate
HBV	Hepatitis B Virus
AUC	Area Under Curve
ROC	Receiver Operating Characteristics
